# Early vasopressin infusion improves oxygenation in infants with congenital diaphragmatic hernia

**DOI:** 10.3389/fped.2023.1104728

**Published:** 2023-03-29

**Authors:** Irma Capolupo, Domenico Umberto De Rose, Francesca Mazzeo, Francesca Monaco, Paola Giliberti, Francesca Landolfo, Alessandra Di Pede, Alessandra Toscano, Andrea Conforti, Pietro Bagolan, Andrea Dotta

**Affiliations:** ^1^ Neonatal Intensive Care Unit, Medical and Surgical Department of Fetus – Newborn – Infant, “Bambino Gesù” Children’s Hospital IRCCS, Rome, Italy; ^2^ Perinatal Cardiology, Medical and Surgical Department of Fetus – Newborn – Infant, “Bambino Gesù” Children’s Hospital IRCCS, Rome, Italy; ^3^ Neonatal Surgery Unit, Medical and Surgical Department of Fetus – Newborn – Infant, “Bambino Gesù” Children’s Hospital IRCCS, Rome, Italy; ^4^ Department of Systems Medicine, University of Tor Vergata, Rome, Italy

**Keywords:** pulmonary hypertension, CDH, neonates, hypotension, oxygenation index, near-infrared spectroscopy, NIRS

## Abstract

**Objective:**

Congenital Diaphragmatic Hernia (CDH) is a complex disease including a diaphragmatic defect, lung hypoplasia, and pulmonary hypertension. Despite its increasing use in neonates, the literature on the use of vasopressin in neonates is limited. The aim of this work is to analyze the changes in clinical and hemodynamic variables in a cohort of CDH infants treated with vasopressin.

**Methods:**

Among CDH infants managed at the Neonatal Intensive Care Unit (NICU) of our hospital from May 2014 to January 2019, all infants who were treated with vasopressin, because of systemic hypotension and pulmonary hypertension, were enrolled in this retrospective study. The primary outcome was the change in oxygenation index (OI) after the start of the infusion of vasopressin. The secondary outcomes were the changes in cerebral and splanchnic fractional tissue oxygen extraction (FTOEc and FTOEs) at near-infrared spectroscopy, to understand the balance between oxygen supply and tissue oxygen consumption after the start of vasopressin infusion. We also reported as secondary outcomes the changes in ratio of arterial oxygen partial pressure (PaO2) to fraction of inspired oxygen (FiO2), heart rate, mean arterial pressure, serum pH, and serum sodium.

**Results:**

We included 27 patients with isolated CDH who received vasopressin administration. OI dramatically dropped when vasopressin infusion started, with a significant reduction according to ANOVA for repeated measures (*p* = 0.003). A global significant improvement in FTOEc and FTOEs was detected (*p* = 0.009 and *p* = 0.004, respectively) as a significant reduction in heart rate (*p* = 0.019). A global significant improvement in PaO2/FiO2 ratio was observed (*p* < 0.001) and also at all time points: at 6 h since infusion (*p* = 0.015), 12 h (*p* = 0.009), and 24 h (*p* = 0.006), respectively. A significant reduction in sodium levels was observed as expected side effect (*p* = 0.012). No significant changes were observed in the remaining outcomes.

**Conclusion:**

Our data suggest that starting early vasopressin infusion in CDH infants with pulmonary hypertension could improve oxygenation index and near-infrared spectroscopy after 12 and 24 h of infusion. These pilot data represent a background for planning future larger randomized trials to evaluate the efficacy and safety of vasopressin for the CDH population.

## 1. Introduction

Congenital diaphragmatic hernia (CDH) is a severe birth defect that affects 1 in 2,500 live births, with the diaphragm defect leading to herniation of abdominal contents into the thorax. Despite improvements in prenatal and postnatal care and survival to discharge (1), this condition is still associated with high mortality and morbidity caused by pulmonary hypoplasia and pulmonary hypertension (PH). The perinatal stabilization period is a critical moment in which PH may cause hypoxemic respiratory failure and shock. Its management is a major target in CDH infants: some authors reported that the incidence of PH in CDH is upwards of 85% (2).

Vasopressin is a non-adrenergic vasopressor that acts as a systemic vasoconstrictor and as a selective pulmonary vasodilator, with minimal chronotropic effects and a lack of adverse inotropic action on the heart. Despite its increasing use in neonates, the literature on the use of vasopressin (VP) in neonates is limited (3–5). Acker et al. already described that vasopressin improved hemodynamic status in 13 infants with CDH and catecholamine-resistant refractory hypotension (6). The aim of this work is to analyze the changes in clinical and hemodynamic variables in a cohort of infants with CDH treated with vasopressin infusion.

## 2. Materials and methods

### 2.1. Population and design of the study

Among CDH infants managed at the Neonatal Intensive Care Unit (NICU) of “Bambino Gesù” Children's Hospital (Rome, Italy) from May 2014 to January 2019, all infants who were treated with vasopressin because of systemic hypotension and pulmonary hypertension were enrolled into this retrospective study. We excluded preterm infants born before 34 weeks of gestational age and those with major congenital malformations associated.

During the study period, CDH patients were managed according to defined protocols (CDH EURO Consortium Consensus) (7). Our local first-line treatment included milrinone and dopamine. Vasopressin was used as a rescue when dopamine was not sufficient to guarantee adequate systolic pressure. Vasopressin was administered to guarantee hemodynamic stability up to surgical repair and then gradually decreased.

In the case of persistent pulmonary hypertension, we added an intravenous phosphodiesterase type 5 inhibitor (sildenafil) after about 24 h since the start of vasopressin.

After surgical repair, if there was no or an insufficient response to iNO, intravenous prostacyclin or medication involving the endothelin pathway has been considered (7).

Data of patients were obtained from electronic medical records.

First, we recorded their demographic and clinical characteristics (sex, gestational age, birth weight, prenatal diagnosis, Lung area to Head circumference Ratio Observed/Expected in children with prenatal diagnosis, delivery mode, Apgar at 5 min of life, etc…).

In order to verify the hemodynamic instability of these infants, we recorded the use of vasopressin (hours of life at the start of infusion and maximum dose that was used), hemoglobin level at the start of vasopressin infusion, use of milrinone (hours of life at the start of infusion and maximum dose that was used), use of inhaled nitric oxide (iNO), use of further drugs for pulmonary hypertension (sildenafil, bosentan, epoprostenol), use of vasoactive drugs (dopamine, dobutamine, epinephrine, norepinephrine) and maximum vasoactive inotropic score, use of hydrocortisone, initial ventilation strategy used (conventional mechanical ventilation or high-frequency oscillatory ventilation), use of ECMO, hours between birth and surgical repair.

Therefore, we recorded changes in Clinical and Hemodynamic Variables before vasopressin infusion (time point 0, T0) and after 6 h (time point 1, T1), 12 h (time point 2, T2), and 24 h from the start of infusion (time point 3, T3).

The primary outcome was the change in oxygenation index (OI) after the start of vasopressin infusion.

The secondary outcomes include changes in near-infrared spectroscopy after the start of vasopressin infusion. A near-infrared spectrometer (NIRS; Invos 5100—Somanetics Corp, Troy, Michigan) equipped with two independent emittent-sensor pairs was used for simultaneous measurement of regional cerebral and splanchnic tissue oxygen saturation (rSO_2_c and rSO_2_s, respectively) in all infants, in order to understand the balance between oxygen supply and tissue oxygen demand. Cerebral and splanchnic fractional tissue oxygen extraction (FTOE) was calculated using the following formula [FTOE = (SaO_2_ – rSO_2_)/SaO_2_ ], and changes after the start of vasopressin infusion were also evaluated (8).

We also reported as secondary outcomes the changes in the ratio of arterial oxygen partial pressure (PaO2) to the fraction of inspired oxygen (FiO2), heart rate, mean arterial pressure, serum pH, and serum sodium. PaO2 assessments were from radial artery punctures.

Standard clinical echocardiography was performed before and within 24 h after vasopressin infusion to assess mean pulmonary arterial pressure and to compare it with systemic systolic pressure, defining it as <2/3 systemic systolic pressure (mild PH), ≥2/3 systemic systolic pressure (moderate PH), or systemic-to-suprasystemic pressure (severe PH) (9). The direction of the shunt through the patent ductus arteriosus and the eventual reversal of flow after vasopressin infusion (as an indirect sign of a lowered pulmonary arterial pressure) were also recorded.

### 2.2. Ethical approval and statistical analysis

This preliminary study reported only a retrospective analysis of data available through the Institutional Database. Personal data were restricted to essential information and were treated in order to guarantee the respect of the privacy of the involved patients, as specifically stated by Italian Law D.Lgs n.196 of 2003 about personal data protection. Therefore, the study did not require preliminary evaluation by the local Ethical Committee.

Data are presented as numbers and percentages for categorical variables, comparing them with the Fisher test. Continuous variables are expressed as mean ± standard deviation (SD) if they were normally distributed or as median and interquartile range if normality could not be accepted, according to the D'Agostino-Pearson test. Global changes in clinical and hemodynamic variables after vasopressin infusion were compared with analysis of variance (ANOVA) for repeated measures with a Huynh-Feldt correction (when epsilon is greater than 0.75) or Greenhouse-Geisser correction (preferred when epsilon <0.75). Conversely, pairwise comparisons between different time points were analyzed with a Bonferroni post-hoc test. A *p*-value <0.05 was considered significant. Statistical analysis was performed using software programs Microsoft Excel (2016 for Windows) and MedCalc (version 12.7 for Windows).

## 3. Results

In this retrospective study, we evaluated the medical records of 70 CDH infants born during the study period. After excluding two patients born before 34 weeks GA and 20 patients with major congenital malformations, we initially considered for inclusion in this study 48 patients with isolated CDH. Among these, 21 patients in whom vasopressin infusion was not administered were also excluded: of these 21, fifteen survived (71.4%), four required patch repair (19.0%), four required other inotropes and ECMO (19.0%), and two received iNO (9.5%).

**Figure 1:**
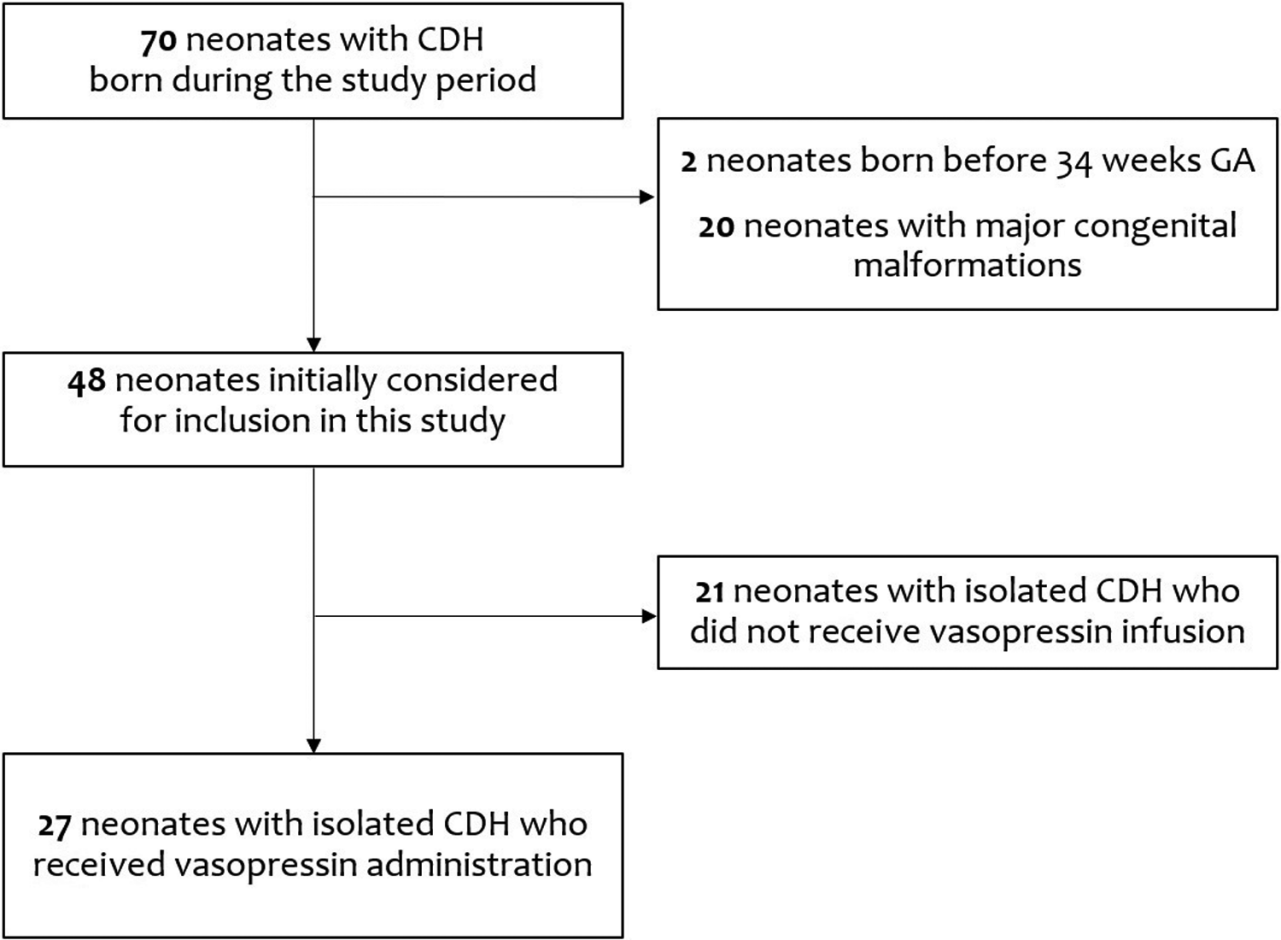
Flow-chart of the design of the study.

Therefore, we analyzed data from 27 patients with isolated CDH who were managed with vasopressin administration (Figure 1).

We reported the demographic and clinical characteristics of included infants in Table 1. Of these 27 patients, 19 survived (70.4%), whereas 7 infants died because of pulmonary hypertension and 1 of sepsis. On the first day of life, echocardiographic evaluation displayed a patent ductus arteriosus in 24 infants (88.9%) and an interatrial communication in 16 infants (59.3%); two infants (7.4%) had a small ventricular septal defect (VSD) not requiring surgery. Three infants of this cohort (11.1%) underwent Extra-Corporeal Membrane Oxygenation (ECMO) on the first day of life.

**Table 1:** Characteristics of CDH infants included in the study. Results are expressed as mean ± standard deviation, median (IQ range), and numbers (percentage).

Characteristics	CDH patients (*n* = 27)
Males	11 (40.7%)
Gestational age (weeks)	37.3 ± 1.6
Late preterm (34–36 weeks)	7 (25.9%)
Birth weight (grams)	2890 ± 495
Prenatal diagnosis	27 (100%)
Lung area to Head circumference Ratio (Observed/Expected) in children with prenatal diagnosis	42.6 ± 13.1
Cesarean section	24 (88.9%)
Apgar at 5 min	8 (8 / 9)
Outborn	17 (73.9%)
Survived	19 (70.4%)
Left-side hernia	22 (81.5%)
Liver herniation	15 (55.6%)
HFOV as starting ventilation strategy	10 (37.0%)
McGoon index	1.37 (1.26 / 1.60)
**Defect size**	
- A	0
- B	8 (29.6%)
- C	9 (33.3%)
- D	4 (14.8%)
- died before surgical repair and defect identification	6 (22.3%)
Patch repair	12/21 (57.1%)
Age at surgery (hours of life)	76 (62.5 / 102)
Maximal vasoactive inotrope score (median, IQR)	31 (20.8–63.5)
Extracorporeal membrane oxygenation	3 (11.1%)
Length of stay of survived infants (days)	51 (46 / 73)

Only in 2 infants (7.4%) we observed a left ventricular dysfunction at echocardiographic evaluation. However, 22 infants (81.5%) received milrinone, starting at a median of 9 h (IQR 3-29). Twenty-six infants (96.3%) needed dopamine, four infants (14.8%) dobutamine, six infants (22.2%) epinephrine, and nine infants (33.3%) norepinephrine. Twelve infants (44.4%) were managed using inhaled nitric oxide (iNO): of them, all infants who died previously received iNO treatment. All infants also received hydrocortisone to maintain adequate blood pressure, sparing inotropes.

We treated all 27 infants with vasopressin, starting infusion at a median of 12 h (IQR 7-30). The mean level of hemoglobin at the start of vasopressin infusion was 15.9 ± 2.1 g/L. Vasopressin was administered for about 72 h in most patients.

**Figure 2:**
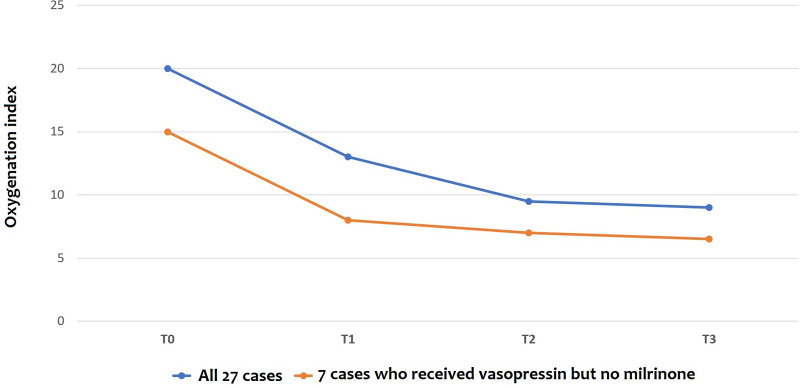
Changes in oxygenation Index (OI) before and after vasopressin infusion: the line shows the trend of median values. *T0: before vasopressin infusion; T1: after 6 h from the start of infusion; T2: after 12 h from the start of infusion; T3: after 24 h from the start of infusion*.

The oxygenation index (OI) dramatically dropped when vasopressin infusion started, as shown in Figure 2, with a significant reduction according to ANOVA for repeated measures with a Huynh-Feldt correction (*p* = 0.003) both in infants receiving milrinone and vasopressin and in infants receiving vasopressin only. Pairwise comparisons between OI values at T0 and OI values at T1, T2, and T3 showed that there were significant differences found after 12 h and 24 h of infusion (*p* = 0.027 and *p* = 0.043, respectively), but not after the first six hours of infusion (*p* = 0.132).

A global significant improvement in FTOEc and FTOEs was detected (*p* = 0.009 and *p* = 0.004, respectively) as a significant reduction in heart rate (*p* = 0.019).

A global significant improvement in PaO2/FiO2 ratio was observed (*p* < 0.001) and also at all time points: at T1 (*p* = 0.015), T2 (*p* = 0.009), and T3 (*p* = 0.006), respectively.

A significant reduction in sodium levels was observed as an expected side effect (*p* = 0.012). No significant changes were observed in the remaining outcomes (Table 2).

**Table 2:** Changes in clinical and hemodynamic variables.

	T0 (before vasopressin)	T1 (after 6 h from the start of vasopressin)	T2 (after 12 h from the start of vasopressin)	T3 (after 24 h from the start of vasopressin)	*p*-value
Oxygenation index	20 (12.5 / 35.8)	13 (6 / 41)	9.5 (5.8 / 25.3)	9 (5 / 24.5)	***p* = 0.003**
Cerebral fractional tissue oxygenation extraction (FTOEc)	0.274 ± 0.130	0.203 ± 0.103	0.221 ± 0.096	0.176 ± 0.086	***p* = 0.009**
Splanchnic fractional tissue oxygenation extraction (FTOEs)	0.328 ± 0.183	0.219 ± 0.178	0.208 ± 0.133	0.167 ± 0.111	***p* = 0.004**
PaO2/FiO2	68.0 (43.3–123.3)	144.5 (41.5–197.5)	173.0 (72.5–204)	168.0 (83.0–214.2)	***p* < 0.001**
Heart rate (beats per minute)	146 ± 19	141 ± 24	131 ± 19	132 ± 19	NS
Mean arterial pressure (mmHg)	41 (36–49)	44 (38–45)	47 (41–52)	47 (42–54)	NS
FiO2 (%)	40 (30–100)	45 (23–100)	30 (25–60)	30 (23–50)	NS
Serum pH	7.33 ± 0.11	7.35 ± 0.14	7.35 ± 0.12	7.39 ± 0.09	NS
Serum sodium (mmol/L)	136 ± 3	135 ± 4	133 ± 5	132 ± 6	***p* = 0.012**

*Results are expressed as mean ± standard deviation, median (IQ range), and numbers*. NS, not statistically significant.

Hyponatremia was managed in all 18/27 infants (66.7%) with fluid restriction: of them, 16/18 (88.9%) also received a careful supplementation of serum sodium during vasopressin administration.

**Figure 3:**
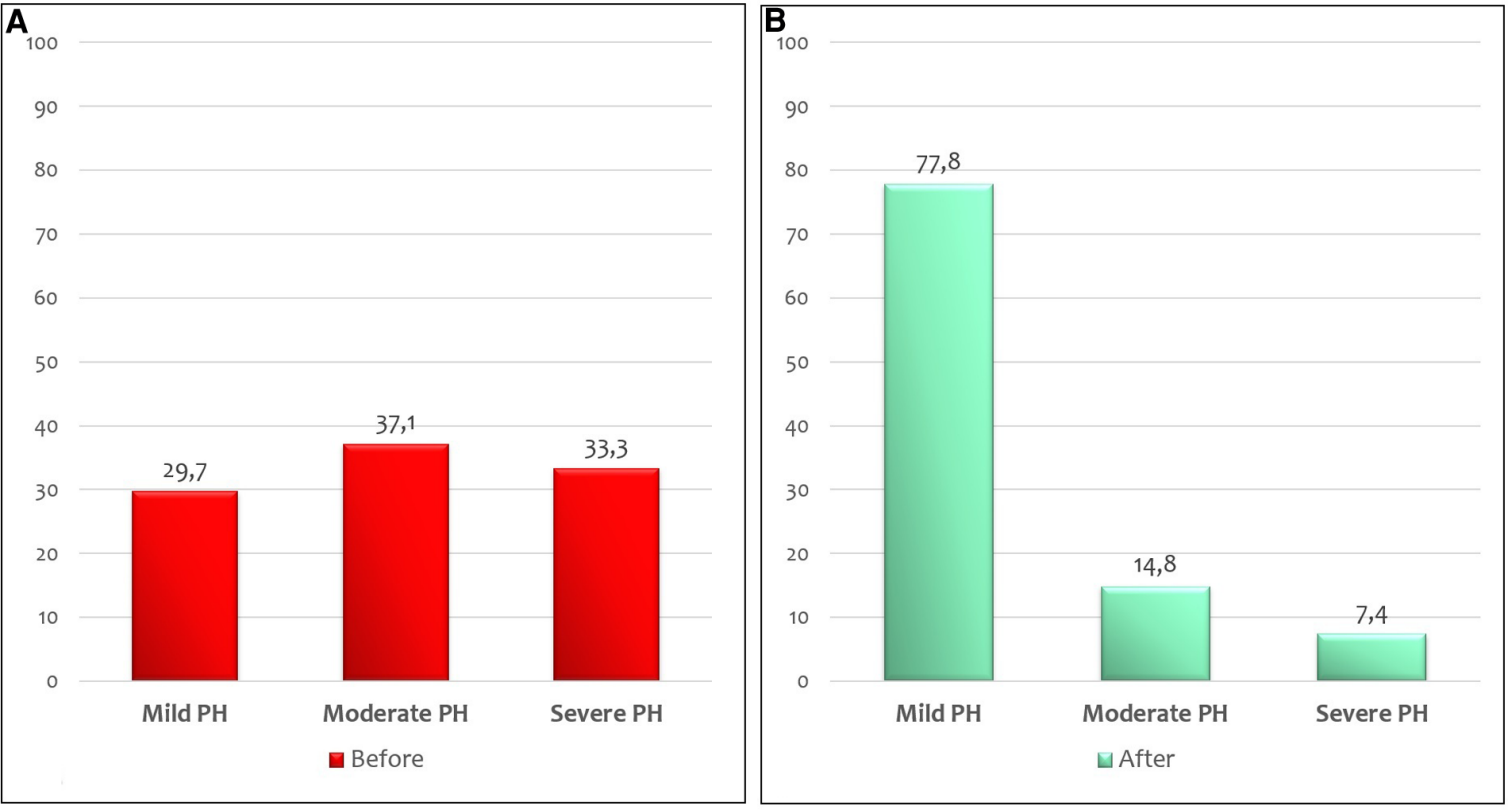
Percentage of CDH infants with mild, moderate, and severe PH before (**A**) and after (**B**) 24 h of vasopressin infusion at echocardiographic evaluation.

At the echocardiographic evaluation, a significant reduction in the percentage of infants with moderate-to-severe pulmonary arterial pressure was detected after vasopressin infusion (*p* = 0.004) (Figure 3). None of the patients managed with vasopressin showed a worsening of PH. In 19/21 infants (90.5%) with a bidirectional shunt or a right-to-left shunt, a reversal of ductal flow was observed after vasopressin infusion.

**Figure 4:**
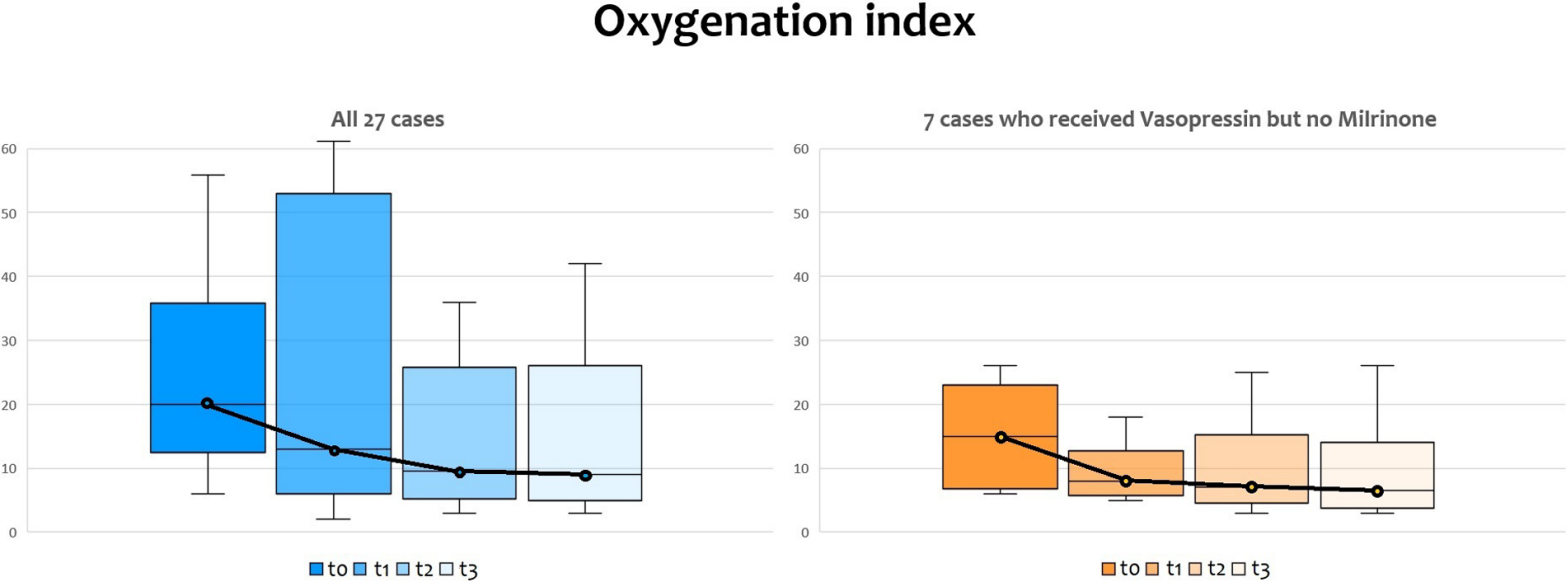
Changes in oxygenation index (OI) before and after vasopressin infusion, with medians and IQR bars. T0: before vasopressin infusion; T1: after 6 h from the start of infusion; T2: after 12 h from the start of infusion; T3: after 24 h from the start of infusion.

Intravenous sildenafil was added in 15 patients (55.6%) after about 24 h since the start of vasopressin infusion because of clinical signs of persistent pulmonary hypertension.

After surgical repair, if there was no or an insufficient response to iNO, or if there was a left ventricular dysfunction contraindicating the use of iNO, 11 patients (40.7%) still had clinical signs of persistent pulmonary hypertension and were managed with other drugs (three patients received epoprostenol and bosentan; four patients only epoprostenol; four patients only bosentan).

## 4. Discussion

To our knowledge, this is the largest cohort study of vasopressin use in the setting of pulmonary hypertension including only infants with CDH to date. Newborns are not small children or adults, with special concerns regarding drug therapy and significant changes in the pharmacokinetics and pharmacodynamics of many drugs (10). Given the difficulty of performing a randomized trial about drugs in rare disorders such as CDH during neonatal age, and reaching adequate sample sizes to build a correct trial, neonatologists often use off-label drugs in acute situations outside of a controlled trial to have a further opportunity to avoid ECMO and death. Considering the increasing use of vasopressin after cardiac surgery in children, we started using vasopressin in CDH infants in 2014. Furthermore, in 2014 Acker et al. already reported that vasopressin improves systemic hemodynamics without adverse effects on pulmonary hemodynamics in a subset of 13 infants with CDH with catecholamine-resistant hypotension (6).

Hemodynamic instability, which is common in CDH newborns, especially during the transition in the first hours of life, necessitates early identification, interpretation of the etiology, and efficient therapy to maximize perfusion and mitigate the symptoms of severe PH (11). Because the left ventricle may be smaller and less compliant, fluid resuscitation must be used with caution to avoid pulmonary edema; the absence of a quick response to fluid typically indicates the need for inotropic support (7). In particular, intravenous milrinone is now a commonly used medication in CDH infants because of its vasodilator and inotrope effects (12).

Indeed, almost every infant born with CDH will have some degree of PH, with a prevalence that decreases substantially in the first weeks of life, and the majority showing a resolved PH by discharge (13). Examining CDH survivors, Dillon et al. reported that one-third of them had pulmonary-to-systemic artery pressure ratios beyond three weeks of treatment (14). Interestingly, Keller et al. described how endothelin-1 (ET1) has a potential role in the pathobiology of infants with severe CDH: plasma ET1 levels at 1 and 2 weeks were higher in infants with poor outcomes compared with infants discharged on room air (15). Thus, therapies that modulate ET1 and its effectors could be a possible treatment for these infants, but the road is still long. Indeed, the management of PH is still challenging, and in clinical routine physicians use PH-targeted drugs that are still off-label in the neonate.

In our cohort, 29.6% of infants with PH died. This result is in line with observed survival rates for CDH neonates with PH (20%–45%) during the first year (16).

The most widely used medication in neonatal PH management is inhaled nitric oxide (iNO) (17). iNO can improve oxygenation and reduce the acute need for ECMO in neonates with other causes of PH, but not definitively in CDH (18–21).

Most CDH patients (92.5%) who required ECMO received iNO prior to or not on the same day as ECMO, among a large international cohort: the treatment with iNO was associated with a 15% higher absolute mortality rate (22). We confirm this trend, considering that all infants who died in this cohort previously needed iNO treatment.

In our cohort, vasopressin was started early and not only after patients met the criteria for initiation of ECMO, as in Acker's report (6). We started vasopressin infusion at a median of 12 h after the onset of systemic hypotension and following an echocardiographic confirmation of PH, with a range of 0.0003 and 0.001 units/kg/min and without boluses. Only three patients (11.1%) required ECMO, although vasopressin infusion among our patients: this low number is partly due to the availability of ECMO in our hospital only since 2012. Conversely, a trial of vasopressin infusion was started by Acker and colleagues before ECMO therapy in 11 of 13 patients, and only 45.5% of infants did not require ECMO. They did not find a significant improvement in the oxygenation index as in our cohort, possibly due to their smaller sample size, but there was similarly a trend towards improved oxygenation index, especially in the subjects not needing ECMO.

We also observed a global significant improvement in cerebral and splanchnic oxygenation, which should be maintained as stable as possible, considering the modifications in cerebral and splanchnic hemodynamics on near-infrared spectroscopy these children undergo (23). Before the start of vasopressin infusion, NIRS values showed a blood flow centralization, with cerebral tissue oxygenation extraction still within normal limits but associated with high splanchnic FTOE values to compensate (high tissue oxygenation extraction as a response to a low income). During vasopressin infusion, we observed a gradual reduction of splanchnic FTOE up to normal values, whereas cerebral oxygenation was maintained within normal ranges, and this is the main novelty of this study.

Hyponatremia (serum Na < 134 mmol/L) is an expected side effect of vasopressin, which we reported in 66.7% of our infants. These findings are in line with the case series of infants treated with vasopressin described by Budniok et al. (64% of infants): they reported severe hyponatremia (less than 125 mmol/L) in 7 episodes (33%) (3). In our cohort, among 18 infants managed with hyponatremia, we detected only two episodes of severe hyponatremia (11.1%), showing how close control with fluid restriction and careful supplementation of serum sodium is required during vasopressin administration and this side effect can be counterbalanced.

We have several data in favor of the biological plausibility of our findings. Sugawara et al. showed in a rat model of a pulmonary hypertensive crisis that vasopressin, but not phenylephrine or norepinephrine, resulted in better hemodynamic and vascular recovery. Indeed, vasopressin contracted femoral but not pulmonary arteries, whereas phenylephrine contracted both arterial beds. Hence, improvements with vasopressin might be associated with decreased pulmonary vascular resistance and selective systemic vasoconstriction (24). The vasotonic effects of vasopressin are mainly mediated by the activation of V1 vascular receptors (V1Rs) and modulation of ATP-sensitive K channels (KATP). Its vasodilatory actions are mediated by the activation of endothelial oxytocin receptors (OTRs) that trigger the activation of endothelial isoforms of nitric oxide synthase (NOS) (25–28).

The effects of vasopressin are twofold: on the one hand, pulmonary vasodilatation; on the other, systemic vasoconstriction, and this can be useful to guarantee hemodynamic stability in infants with CDH and avoid global derangement. Goals for hemodynamic management include heart rate and blood pressure within the normal range for gestational age, arterial pH > 7.2, urine output >1 ml/kg/h, lactate concentration <3 mmol/L, and adequate end-organ perfusion (7). Individualized hemodynamic management is required in all neonates, but especially in those with CDH. If there are signs suggestive of poor perfusion, volume resuscitation with 10 ml/kg of an isotonic solution should be considered. The treatment should be guided by cardiac function evaluation by echocardiography. Afterward, often vasopressors such as dopamine are needed to increase systemic blood pressure and improve tissue perfusion. Catecholamine-resistant hypotension is a frequent occurrence in CDH for which vasopressin may be effective (29).

Our data support the use of vasopressin in infants with CDH, as previously reported in a case series by Acker et al. who described how vasopressin therapy increased mean arterial pressure and decreased pulmonary/systemic pressure ratio, heart rate, and the fraction of inspired oxygen (6).

Our findings are limited by the small sample size of included infants, the lack of urine output data, and the need for standardized echocardiographic data collected prospectively at different time points due to the retrospective design of the study. Moreover, we don’t have a placebo control group or a comparable time-matched cohort, and therefore we don't have enough data to assess if the amelioration obtained with vasopressin was summatory to that obtained with other drugs or not. A prospective study is needed to fully evaluate the dynamic interplay of the cardiovascular system in these patients.

Interestingly, a recent preprint reported a retrospective evaluation of term infants with persistent pulmonary hypertension under a standardized protocol, which included the use of vasopressin. Santelices et al. included 48 neonates with different disorders: of them, 39 neonates had CDH. They confirmed our findings also by echocardiographic within 6 h prior to the start of vasopressin and between 3 and 24 h from the start of the drug: furthermore, all patients were receiving iNO at 20 ppm. However, their results concern all patients and not only those with CDH (30).

Conversely, we confirmed in a homogenous cohort of CDH infants the positive impact of vasopressin infusion in improving hemodynamic status and oxygenation. These pilot data represent a background for planning future larger studies to evaluate the efficacy and safety of vasopressin for the CDH population.

## 5. Conclusion

Our data suggest that the vasopressin infusion in CDH infants can be useful in reducing pulmonary hypertension since we found a significant reduction in oxygenation index after 12 and 24 h of infusion, with a similar trend in both infants receiving milrinone and vasopressin and in those receiving only vasopressin. Furthermore, we also describe a significant improvement in cerebral and splanchnic oxygenation at near-infrared spectroscopy. However, further randomized studies are needed to confirm our uncontrolled retrospective findings in a multicenter cohort.

## Data Availability

The raw data supporting the conclusions of this article will be made available by the authors, without undue reservation.

## References

[1] Morini F Lally KP Lally PA Crisafulli RM Capolupo I Bagolan P. Treatment strategies for congenital diaphragmatic hernia: change sometimes comes bearing gifts. Front Pediatr. (2017) 5:1–8. 10.3389/fped.2017.00195 28959686 PMC5603669

[2] Wehrmann M Patel SS Haxel C Cassidy C Howley L Cuneo B Implications of atrial-level shunting by echocardiography in newborns with congenital diaphragmatic hernia. J Pediatr. (2020) 219:43–7. 10.1016/j.jpeds.2019.12.037 32014282

[3] Budniok T ElSayed Y Louis D. Effect of vasopressin on systemic and pulmonary hemodynamics in neonates. Am J Perinatol. (2021) 38:1330–4. 10.1055/s-0040-1712999 32485754

[4] Mohamed A Nasef N Shah V McNamara PJ. Vasopressin as a rescue therapy for refractory pulmonary hypertension in neonates: case series. Pediatr Crit Care Med. (2014) 15:148–54. 10.1097/PCC.0b013e31829f5fce 24141655

[5] Mohamed AA Louis D Surak A Weisz DE McNamara PJ Jain A. Vasopressin for refractory persistent pulmonary hypertension of the newborn in preterm neonates–a case series. J Matern Fetal Neonatal Med. (2022) 35:1475–83. 10.1080/14767058.2020.1757642 32349572

[6] Acker SN Kinsella JP Abman SH Gien J. Vasopressin improves hemodynamic status in infants with congenital diaphragmatic hernia. J Pediatr. (2014) 165:53–58.e1. 10.1016/j.jpeds.2014.03.059 24840762 PMC4116488

[7] Snoek KG Reiss IKM Greenough A Capolupo I Urlesberger B Wessel L Standardized postnatal management of infants with congenital diaphragmatic hernia in Europe: the CDH EURO consortium consensus - 2015 update. Neonatology. (2016) 110:66–74. 10.1159/000444210 27077664

[8] Giliberti P Mondì V Conforti A Lombardi MH Sgrò S Bozza P Near infrared spectroscopy in newborns with surgical disease. J Matern Fetal Neonatal Med. (2011) 24:56–8. 10.3109/14767058.2011.607673 21942593

[9] Lusk LA Wai KC Moon-Grady AJ Steurer MA Keller RL. Persistence of pulmonary hypertension by echocardiography predicts short-term outcomes in congenital diaphragmatic hernia. J Pediatr. (2015) 166:251–256.e1. 10.1016/j.jpeds.2014.10.024 25453248 PMC4308510

[10] De Rose DU Cairoli S Dionisi M Santisi A Massenzi L Goffredo BM Therapeutic drug monitoring is a feasible tool to personalize drug administration in neonates using new techniques: an overview on the pharmacokinetics and pharmacodynamics in neonatal age. Int J Mol Sci. (2020) 21:5898. 10.3390/ijms21165898 32824472 PMC7460644

[11] Canadian Congenital Diaphragmatic Hernia Collaborative, Puligandla PS Skarsgard ED Offringa M Adatia I Baird R Diagnosis and management of congenital diaphragmatic hernia: a clinical practice guideline. CMAJ. (2018) 190:E103–12. 10.1503/cmaj.170206 29378870 PMC5790558

[12] Lakshminrusimha S Keszler M Kirpalani H Van Meurs K Chess P Ambalavanan N Milrinone in congenital diaphragmatic hernia – a randomized pilot trial: study protocol, review of literature and survey of current practices. Matern Health Neonatol Perinatal. (2017) 3:27. 10.1186/s40748-017-0066-9 PMC5704584 29209510

[13] Varghese NP Tillman RH Keller RL. Pulmonary hypertension is an important co-morbidity in developmental lung diseases of infancy: bronchopulmonary dysplasia and congenital diaphragmatic hernia. Pediatr Pulmonol. (2021) 56:670–7. 10.1002/ppul.25258 33561308

[14] Dillon PW Cilley RE Mauger D Zachary C Meier A Altman RP The relationship of pulmonary artery pressure and survival in congenital diaphragmatic hernia. J Pediatr Surg. (2004) 39:307–12. 10.1016/j.jpedsurg.2003.11.010 15017543

[15] Keller RL Tacy TA Hendricks-Munoz K Xu J Moon-Grady AJ Neuhaus J Congenital diaphragmatic hernia: endothelin-1, pulmonary hypertension, and disease severity. Am J Respir Crit Care Med. (2010) 182:555–61. 10.1164/rccm.200907-1126OC 20413632 PMC2937245

[16] Sanchez Mejia AA Rodgers NJ. Evaluation and monitoring of pulmonary hypertension in neonates with congenital diaphragmatic hernia. Curr Treat Opt Cardiovasc Med. (2019) 21:11. 10.1007/s11936-019-0711-x 30767157

[17] Sharma M Callan E Konduri GG. Pulmonary vasodilator therapy in persistent pulmonary hypertension of the newborn. Clin Perinatol. (2022) 49:103–25. 10.1016/j.clp.2021.11.010 35209994

[18] Neonatal Inhaled Nitric Oxide Study Group (NINOS). Inhaled nitric oxide and hypoxic respiratory failure in infants with congenital diaphragmatic hernia. Pediatrics. (1997) 99:838. 10.1542/peds.99.6.838 9190553

[19] Clark RH Kueser TJ Walker MW Southgate WM Huckaby JL Perez JA Low-dose nitric oxide therapy for persistent pulmonary hypertension of the newborn. N Engl J Med. (2000) 342:469–74. 10.1056/NEJM200002173420704 10675427

[20] Barrington KJ Finer N Pennaforte T Altit G. Nitric oxide for respiratory failure in infants born at or near term. Cochrane Database Syst Rev. (2017) 1:CD000399. 10.1002/14651858.CD000399.pub3 28056166 PMC6464941

[21] Rafat N Schaible T. Extracorporeal membrane oxygenation in congenital diaphragmatic hernia. Front Pediatr. (2019) 7:1–9. 10.3389/fped.2019.00336 31440491 PMC6694279

[22] Putnam LR Tsao K Morini F Lally PA Miller CC Lally KP Evaluation of variability in inhaled nitric oxide use and pulmonary hypertension in patients with congenital diaphragmatic hernia. JAMA Pediatr. (2016) 170:1188–94. 10.1001/jamapediatrics.2016.2023 27723858

[23] Conforti A Giliberti P Landolfo F Valfrè L Columbo C Mondi V Effects of ventilation modalities on near-infrared spectroscopy in surgically corrected CDH infants. J Pediatr Surg. (2016) 51:349–53. 10.1016/j.jpedsurg.2015.07.021 26342630

[24] Sugawara Y Mizuno Y Oku S Goto T. Effects of vasopressin during a pulmonary hypertensive crisis induced by acute hypoxia in a rat model of pulmonary hypertension. Br J Anaesth. (2019) 122:437–47. 10.1016/j.bja.2019.01.014 30857600 PMC6435915

[25] Russ RD Walker BR. Role of nitric oxide in vasopressinergic pulmonary vasodilatation. Am J Physiol. (1992) 262:H743–7. 10.1152/ajpheart.1992.262.3.H743 1558183

[26] Evora PR Pearson PJ Schaff HV. Arginine vasopressin induces endothelium-dependent vasodilatation of the pulmonary artery. V1-receptor-mediated production of nitric oxide. Chest. (1993) 103:1241–5. 10.1378/chest.103.4.1241 8131474

[27] Thibonnier M Conarty DM Preston JA Plesnicher CL Dweik RA Erzurum SC. Human vascular endothelial cells express oxytocin receptors. Endocrinology. (1999) 140:1301–9. 10.1210/endo.140.3.6546 10067857

[28] Holmes CL Landry DW Granton JT. Science review: vasopressin and the cardiovascular system part 2 - clinical physiology. Crit Care. (2004) 8(1):15–23. 10.1186/cc2338 14975041 PMC420051

[29] Chatterjee D Ing RJ Gien J. Update on congenital diaphragmatic hernia. Anesth Analg. (2020) 131:808–21. 10.1213/ANE.0000000000004324 31335403

[30] Santelices F Masoli D Kattan J Toso A Luco M. Vasopressina as adjunctive therapy in pulmonary hypertension associated with refractory systemic hypotension in term newborns, 01 November 2022, PREPRINT (Version 1). Available at Research Square [10.21203/rs.3.rs-2203038/v1] 38965377

